# Development of Smart Surfaces for Medicine and Biotechnology: Advances in Glass Functionalization through RDRP Techniques

**DOI:** 10.1021/acsbiomaterials.5c00908

**Published:** 2025-07-31

**Authors:** Michał Sroka, Izabela Zaborniak, Paweł Chmielarz

**Affiliations:** Department of Physical Chemistry, Faculty of Chemistry, 69696 Rzeszow University of Technology, al. Powstańców Warszawy 6, Rzeszów 35-959, Poland

**Keywords:** glass, reversible deactivation radical polymerization, functional polymer coatings, hybrid materials, intelligent surfaces

## Abstract

All glass represents a material with extremely high utility potential in the development of biomaterials and research tools. Due to a number of its unique properties, such as chemical inertness, thermal stability, and transparency, it can be used in the preparation of hybrid materials for medicine and biotechnology. Such materials can be obtained by grafting polymer brushes from glass surface by reversible deactivation radical polymerization (RDRP) techniques. This paper provides a literature review of the foregoing advances in the development of glass surface modification concepts using atom transfer radical polymerization (ATRP) and reversible addition–fragmentation chain transfer polymerization (RAFT). These methods are particularly attractive in designing smart coatings because they enable the synthesis of polymers with a well-defined structure and low dispersity. The resulting materials can then serve as antimicrobial surfaces, tools for selective manipulation of cells, and intelligent platforms for creating cell sheets in tissue engineering. Therefore, the idea of glass modification using RDRP techniques appears to be a promising concept for the future in the development of smart materials for various applications.

## Graphical abstract

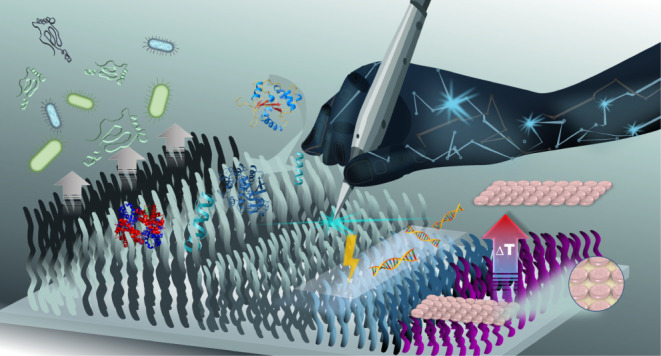

## 1 Introduction

Rapid advances in medicine, biotechnology, and related sciences are associated with increased demand for functional materials. Among them, polymers are attracting special attention due to their favorable mechanical properties and biocompatibility. Many of them play currently a key role in disease therapy and diagnostics,^1^ the development of tissue engineering,^2^ or the design of devices widely used in healthcare systems.^3^ The exploitation potential of polymers can be further enhanced by creating composite or hybrid materials, in which inorganic structural elements are combined with organic macromolecules. In traditional composites, inorganic fillers are dispersed within a polymer matrix.^4^ In contrast, hybrid materials feature organic and inorganic components chemically bonded through either primary or strong secondary interactions. Hybrid materials can be readily produced by functionalizing inorganic substrates with covalently attached polymer chains, known as polymer brushes.^5,6^ Immobilization of polymer brushes on a surface results in a polymeric coating that defines the material’s interaction with its external environment. This surface modification concept has been widely reported for various inorganic substrates, including magnetite,^7^ ZnO,^8^ silica nanoparticles,^9–11^ gold,^12^ titanium,^13^ and silicon wafers,^14–16^ resulting in materials with improved hydrophilicity, biocompatibility, or corrosion resistance.

The properties of hybrid materials frequently depend strictly on the uniformity of the polymer coating bonded to the surface. The morphology of this polymer layer is, in turn, influenced by the length, architecture (linear or branched), and grafting density of the polymer brushes.^17,18^ Therefore, precise control over the polymerization is crucial to obtain materials with the desired properties. Such control cannot be achieved using conventional free radical polymerization. However, surface-initiated reversible deactivation radical polymerization (SI-RDRP) techniques enable the synthesis of polymer brushes with precisely defined molecular weight and low dispersity.^17,19–21^ The three main types of RDRP techniques are atom transfer radical polymerization (ATRP),^22,23^ reversible addition–fragmentation chain transfer (RAFT)^24^ and nitroxide-mediated polymerization (NMP).^25^ Each of these techniques has been successfully applied to the modification of both synthetic and naturally derived materials, allowing for precise tailoring of their inimitable properties.

Among the wide range of inorganic substrates, glass appears to be particularly attractive in the development of hybrid materials with potential applications in medicine and biotechnology.^26^ This is attributed to its widespread use in hospitals, as well as in diagnostic and research laboratories. The eager use of glass in these areas is due to its unique and advantageous properties. Glass is chemically inert, making it an ideal material for the storage and transport of pharmaceuticals, laboratory reagents, and biological samples. Unlike plastics, it does not contain additional organic plasticizers that may leach out under various external conditions. Its high transparency makes it suitable for the production of microscope components and optical lenses. Importantly, for medical applications, glass can be subjected to high temperatures, UV radiation or chemical agents without compromising its structural integrity. This makes it easy to sterilize, a critical factor in clinical environments. Additionally, glass serves as a building component in various biomaterials. Since the development of the first generation of bioglass by Hench et al. at the University of Florida in 1971,^27^ it has been used in the creation of materials and devices for bone and dental surgery,^28^ cancer therapy^29^ or even wound healing.^30^ Despite its widespread use in biomedical applications, there remains considerable potential for the advancement of smart glass-based materials, whose properties and applications have not yet been fully explored.

In recent years, the modification of glass surfaces to develop functional materials has been extensively explored by numerous researchers. This paper presents recent advancements in the fabrication of hybrid materials through the grafting of polymer brushes onto glass surfaces using RDRP techniques. Particular attention is given to the potential applications of these materials in medicine, biotechnology, and tissue engineering. As the use of NMP for glass modification has not yet been reported in the literature, this review will focus exclusively on the idea of using ATRP and RAFT in the preparation of modern glass-based hybrid materials.

## 2 Strategies for Surface Functionalization by RDRP Techniques

Polymers exhibit a wide range of physicochemical properties, largely determined by the type and arrangement of functional groups in their side chains. As a result, applying polymer coatings to various material surfaces can drastically alter properties such as hydrophobicity or hydrophilicity, introduce responsiveness to external stimuli, and influence surface interactions with the surrounding environment. While physical deposition of polymer layers is limited by their week and nonpermanent adhesion, chemical grafting offers a more robust solution by forming mechanically stable coatings through covalent bonding to the substrate. Nevertheless, successful chemical grafting of polymer brushes requires the presence of reactive functional groups on the surface to allow for the immobilization of either polymerization initiator or presynthesized polymer chains.^20,31^ Glass is a particularly suitable substrate for such modifications, as its surface can be easily hydroxylated, for example, through oxygen plasma treatment.^32^ This facilitates straightforward chemical modification, making glass an excellent candidate for hybrid materials using RDRP techniques.

ATRP is currently the most widely used technique among RDRP methods, as it enables the synthesis of polymers with well-defined structure and properties under mild conditions. In ATRP, control over the polymerization is achieved through a dynamic equilibrium between active radicals and dormant chains. This equilibrium requires the presence of a catalyst in the form of a transition metal complex at a lower oxidation state (typically Cu^I^X/L), which reacts with an initiator (an alkyl halide) to form a propagation-capable radical. This reaction simultaneously leads to the oxidation of a metal in the catalytic complex (Cu^II^X_2_/L), serving then as a deactivator of active radicals. By shifting the equilibrium toward the dormant state, the concentration of active radicals is significantly reduced, thereby minimizing unwanted chain termination reactions and promoting uniform chain growth.^33,34^ ATRP is also an attractive method for the synthesis of precisely defined polymers due to the possibility of using a catalyst in low parts-per-million (ppm) concentrations. This is made possible by incorporating a reducing agent that continuously regenerates the active catalyst.^35^ Various approaches have been developed for this purpose, including using chemical agents such as ascorbic acid in activators regenerated by electron transfer ATRP (ARGET ATRP),^36,37^ zerovalent metal in supplemental activator and reducing agent ATRP (SARA ATRP)^38–40^ or radical initiator in initiators for continuous activator regeneration ATRP (ICAR ATRP).^41^ Catalyst regeneration can also be accomplished through physical stimuli, such as electric current in electrochemically mediated ATRP (*se*/*e*ATRP)^42–45^ or light in photoinduced ATRP (*photo*ATRP)^46–48^ and metal-free ATRP.^49–51^

Surface modification using surface-initiated ATRP (SI-ATRP) techniques can be carried out through three distinct manners, as illustrated in Figure 1a–c.^6,52,53^ In the *grafting from* approach, the initiator is covalently bonded to the substrate, typically through an esterification or amidation reaction. This enables the controlled growth of polymer chains directly from the surface and is especially suitable when a high grafting density of polymer brushes is desired for efficient surface modification. In the *grafting through* technique, the monomer unit is immobilized on the substrate surface to form macromonomers, which are then copolymerized with low molecular weight monomers. Although polymer chains also grow directly from the surface in this method, it often results in polymer brushes with relatively low grafting density. A similar drawback characterizes the *grafting onto* approach. This method involves the attachment of presynthesized polymer chains to the surface through a reaction between functional groups at the ends of the polymer chains and those present on the surface. Covalent bonding is most commonly achieved by click chemistry, particularly through copper-catalyzed azide–alkyne cycloaddition (CuAAC).^54^ However, this method typically leads to lower grafting densities due to steric hindrance and diffusion limitations. Despite this drawback, the *grafting onto* approach remains an attractive strategy for surface modification, as it is the only approach that allows full characterization of polymer brush properties before their attachment to the surface. In contrast to ATRP, RAFT belongs to the group of degenerative transfer radical polymerization techniques. RAFT shares several features with conventional free radical polymerization, such as irreversible termination and the use of an external radical source, typically a thermal initiator. The generated radicals are evenly distributed among all growing polymer chains, which promotes uniform propagation kinetics and contributes to a narrow molecular weight distribution. Control over the polymerization in RAFT is primarily governed by a chain transfer agent (CTA), the key component of this technique. The CTA contains the thiocarbonylthio group flanked by two substituents: the R-group and the Z-group. These groups play a critical role in determining the reactivity of the CTA with propagating chains and its ability to form a reversible intermediate that fragments to release a carbon-centered radical.^55,56^ Since RAFT polymerization requires both a radical initiator and CTA, surface-initiated RAFT polymerization (SI-RAFT) can be achieved by immobilizing either the initiator or the CTA on the surface of the substrate (Figure 1d,e). Previous studies have shown that using a surface-tethered initiator leads to a simultaneous reduction in polymer brush thickness and molecular weight of polymers in solution as the concentration of CTA increases.^57^ In addition, this method is often associated with lower initiation efficiency and consequently broader molecular weight distributions of synthesized polymers.^58^ A more commonly adopted strategy for SI-RAFT involves immobilizing the CTA on the surface while keeping a free initiator in solution. CTA can be attached to the surface through either the R-group or Z-group.^59^ When the R-group is anchored to the surface, polymer chains grow directly from it by reacting with monomers and CTA in the solution, typically resulting in high grafting densities of polymer brushes. Nevertheless, this setup increases the risk of radical termination at the surface as the polymerization progresses. This issue can be mitigated by anchoring the CTA via the Z-group, though this often results in a lower grafting density of the polymer brushes.^58^

**Figure 1:**
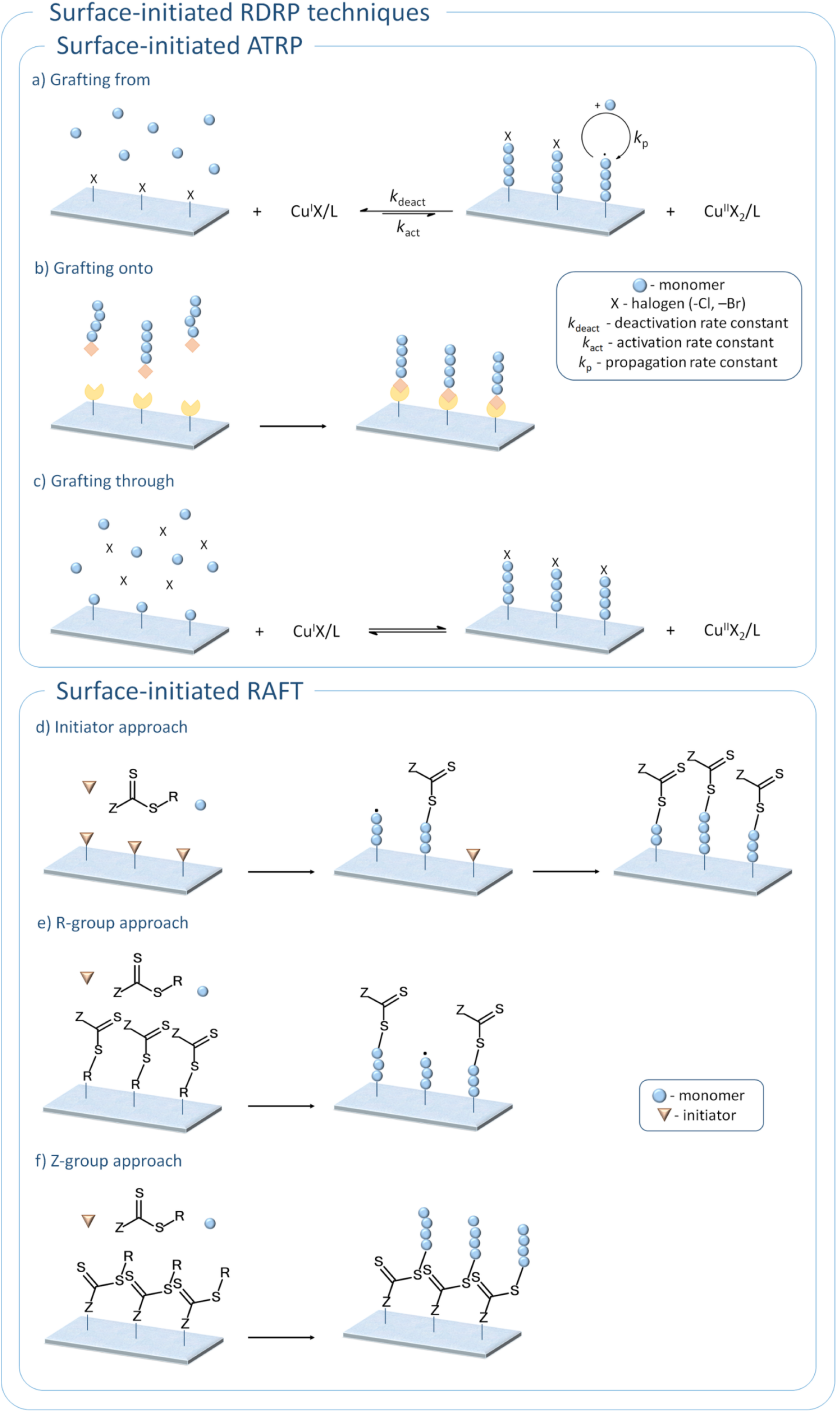
General scheme of surface modification via ATRP^60^ and RAFT.^58^

## 3 Antiadhesive and Antibacterial Surfaces for Medical Applications

The adsorption of organic molecules and bacterial cells on various flat or porous surfaces presents a significant challenge across multiple fields, especially in diagnostics and therapeutics. This phenomenon often leads to so-called “biomaterial-centered infections” (BCI), which arise from biofilm formation on medical devices that remain in direct contact with body fluids or mucous membranes (e.g., cardiovascular implants, urea stents, etc.). BCI compromise the safety of medical instruments and materials that are frequently essential to the proper function of individual organs or the entire organism.^61^ Equally concerning is the formation of thrombi, resulting from the adsorption of fibrinogen and von Willebrand factor (vWF) on materials used in the treatment of cardiovascular diseases. These thrombi pose serious, life-threatening risks to patients.^62^ Given these challenges, there is an urgent need to develop functional coatings that effectively prevent the adhesion of biological entities to materials intended for medical applications.

To develop effective coatings, it is essential to comprehend how to control the adhesion of macromolecules and cells to surfaces. The full mechanism of bacteria adsorption on various materials is not yet fully understood. However, studies suggest that the strength and type of interaction between cells and surfaces strongly depend on the physicochemical properties of the material and the presence of specific receptors on the bacterial membrane. Additionally, biofilm formation can be promoted by the prior accumulation of organic and inorganic compounds, driven by electrostatic interactions and van der Waals forces between molecules and the surface.^63^ To mitigate this issue, poly­(ethylene glycol) (PEG)-based coatings have been extensively used due to their high hydrophilicity and ability to bind water. The formation of a highly hydrated polymer layer tends to generate a repulsive osmotic force, preventing the adsorption of cells and molecules.^64^ However, PEG-based materials are not suitable for medical device applications, as PEG rapidly autooxidizes under physiological conditions in the human body.^65^ Thus, a key challenge lies in identifying hydrophilic materials with significantly greater biostability. A promising alternative is the use of ionic polymers, which affect bacterial cells through electrostatic interactions. Positively charged chains can disrupt bacterial cell membranes’ integrity, while anionic polymers prevent adhesion due to the mutual repulsion between negatively charged chains and the negatively charged bacterial surface.^66^ This approach appears particularly advantageous when compared to coatings containing biocidal agents (e.g., antibiotics). Unlike biocidal coatings, polymeric coatings do not contribute to environmental contamination with antimicrobial substances or promote antibiotic resistance in bacteria. Both hydrophilic and ionic polymers have already been employed for glass surface modification, leading to the development of antifouling coatings with significant potential for medical applications (Figure 2a).

**Figure 2:**
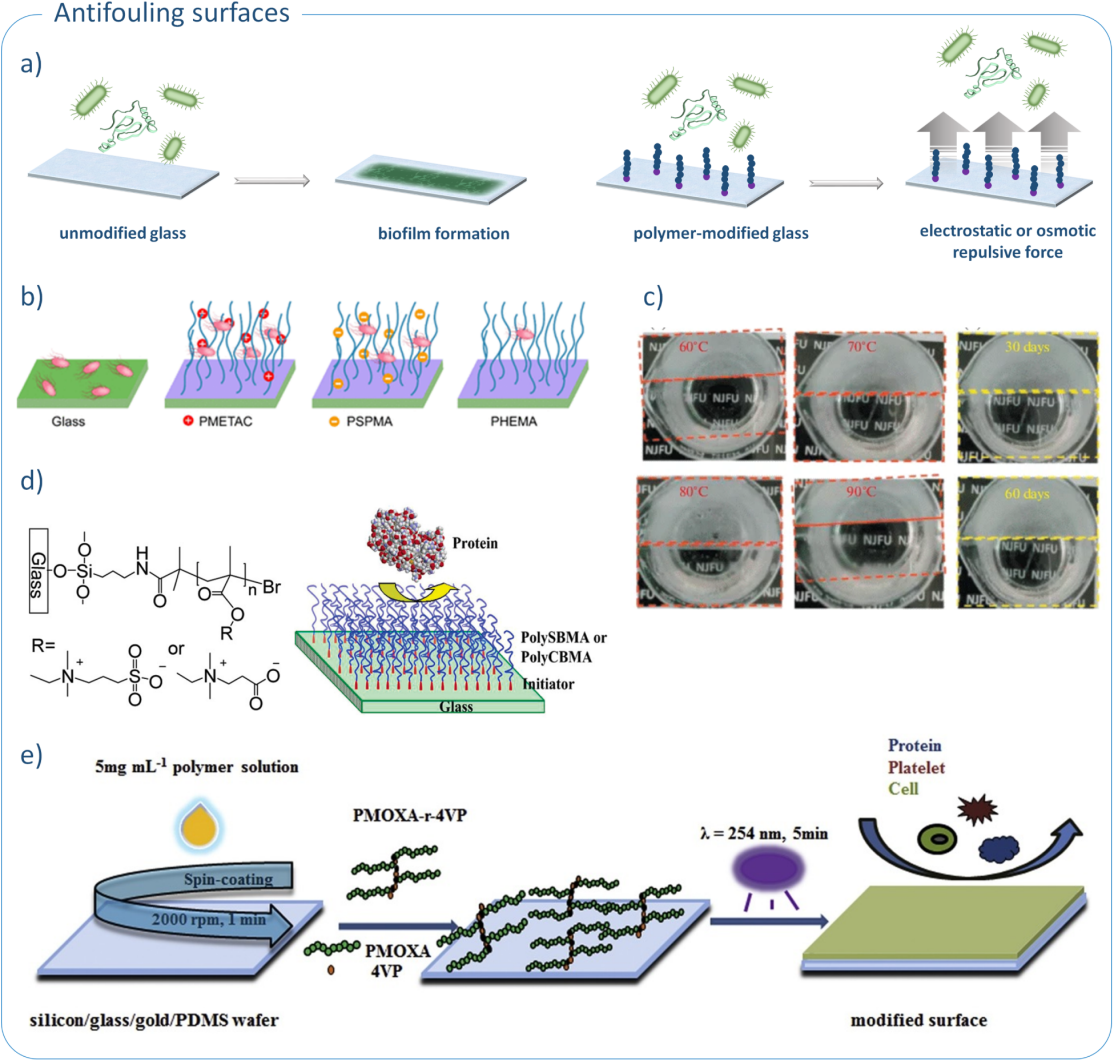
Antifouling properties of polymer-modified glass: a) inhibition of biofilm formation by grafting polymer brushes from glass surface—general mechanism; b) effect of polymer brush charge on bacterial adhesion; reproduced with permission from ref. 67. Copyright 2019, American Chemical Society; c) antifogging properties of glass modified with NAGA/NAG hydrogel; reproduced with permission from ref. 70. Copyright 2021, Pleiades Publishing, Ltd.; d) limited protein adsorption on PSBMA and PCBMA-modified glass surface; reproduced with permission from ref. 74. Copyright 2006, American Chemical Society; e) antifouling performance of PMOXA-*r*-4VP-based coatings; reproduced with permission from ref 78. Copyright 2017, Elsevier B.V.

The impact of the electrostatic charge of the polymer coating on its antiadhesive properties has been extensively investigated by Oh et al. (Figure 2b).^67^ The authors utilized SI-ATRP to prepare three types of homopolymer brushes grafted from the glass surface—cationic poly­[2-(methacryloyloxy)­ethyl trimethylammonium chloride] (PMETAC), neutral poly­(2-hydroxyethyl methacrylate) (PHEMA), and anionic poly­(3-sulfopropyl methacrylate potassium salt) (PSPMA) (Table 2, entry 1). The resulting coatings were uniform and smooth with a similar grafting density (approximately 0.08–0.09 chains/nm^2^) and “dry” thickness of around 50 nm. To evaluate the antibacterial properties of the resulting materials, glass coverslips, both polymer-modified and nonmodified, were incubated with an suspension. The strength of the interactions between the surfaces and cells was assessed by AFM-based single-cell force spectroscopy (SCFS), while relative bacterial adhesion was quantified via an epi-fluorescence microscope. A notable increase in adhesion was observed on PMETAC-coated surfaces after 12 and 24 h of incubation. This can be explained by the occurrence of attractive interactions between the positively charged polymer chains and the negatively charged cell membrane of . Additionally, cationic polymer brushes tended to absorb substances from the culture medium, further reducing their biocidal capabilities. However, PMETAC still possesses the ability to reduce cell adhesion due to its high chain mobility and ease of hydration. This creates an unfavorable environment for bacterial adsorption, resulting in lower cell adhesion on PMETAC-modified glass compared to the unmodified surface. Exceedingly better antifouling properties were achieved for the PSPMA and PHEMA layers. SCFS measurements revealed that the bacterial cells exhibited nonadhesive interactions with the PSPMA and PHEMA coatings, while they adhered strongly to the PMETAC layer. The negatively charged PSPMA chains prevent cell attachment by generating a repulsive force against the negatively charged bacterial surfaces. In addition, anionic polymer brushes can reduce bacterial motility, further limiting biofilm formation. However, negatively charged coatings are not a universal solution for preventing cell adhesion. Some bacteria species, such as , can orient themselves vertically toward the surface, limiting their area with the polyanionic layer and thereby overcoming repulsive forces. The use of neutral polymers, such as PHEMA, eliminates such inconveniences associated with the ionic coatings. Among the tested coatings, PHEMA brushes exhibited the highest water absorption capacity, with a swelling ratio equal to 3.38, compared to 2.56 for PMETAC and 2.34 for PSPMA. This enhanced hydration generates a strong repulsive osmotic force, effectively preventing bacterial adhesion. In addition, neutral polymer chains can also exert repulsive effects on bacterial species that possess cationic ligands or functional groups on their surfaces. Furthermore, PHEMA coatings demonstrated the highest long-term stability of antifouling properties, a crucial factor for biomedical applications.^67^

The use of PHEMA for surface modification has attracted particular interest due to the presence of reactive hydroxyl groups in its side chains. This enables the synthesis of macromolecules with more complex architectures, such as polymer bottlebrushes.^15^ Compared to linear configurations, these branched structures often demonstrate enhanced properties, as they allow for the grafting of side chains, introducing new functionalities to the coating. Jiang et al. obtained polymer bottlebrushes on a glass surface through sequential modification using SI-ATRP technique (Table 2, entry 2).^68^ In the first step, polymerization of HEMA was performed, followed by esterification of hydroxyl groups with α-bromoisobutyryl bromide (BiBB). This layer was then developed by polymerizing 2-(methacryloyloxy)­ethyl choline phosphate (MCP), initiated from brominated PHEMA side chains. Zwitterionic materials, such as polymers containing choline phosphate (CP), have been previously reported to exhibit strong resistance to protein adsorption. However, their ability to prevent bacterial adhesion has not been thoroughly investigated.^69^ To evaluate antifouling properties of different types of coatings, the authors synthesized not only the PHEMA-*g*-PMCP system but also linear PHEMA and PMCP brushes for comparison. Bovine serum albumin (BSA) and fibrinogen were utilized as model proteins to evaluate the protein-resistant characteristics of the modified surfaces. It has been demonstrated that surface modified with PHEMA-*g*-PMCP bottlebrushes exhibited superior antiadhesive properties, with adsorbed fibrinogen and BSA levels of 0.06174 and 0.00782 μg/cm^2^, respectively. In contrast, the surface coated with linear PMCP had higher adsorption levels (0.07497 and 0.01698 μg/cm^2^, respectively). This enhanced protein resistance is attributed to the higher grafting density of CP units in the branched structure, which effectively prevents protein adhesion. Notably, the PCMP-modified surface reduced BSA adsorption by more than 3-fold compared to unmodified glass, confirming the potential of CP-containing polymers for minimizing protein adsorption. To further assess the antifouling properties, bacterial adhesion tests were conducted using and on all surface types. After 72 h of incubation, a significant increase in adsorption of both strains was observed on unmodified glass, suggesting biofilm formation. In contrast, surfaces coated with PHEMA-*g*-PMCP, PHEMA, and PMCP demonstrated a substantial reduction in colony-forming unit (CFU) over the same incubation period. Among the coatings, PMCP demonstrated superior antiadhesive effects against and compared to the PHEMA layer, suggesting that zwitterionic polymers tend to be more efficient in preventing bacterial adsorption. However, PHEMA-*g*-PMCP displayed the most pronounced antibacterial performance, which can be attributed to its higher grafting density of CP units and the synergistic biocidal effect of PHEMA and PMPC. In the last step, the hemocompatibility of modified surfaces was evaluated. The platelet adhesion tests revealed that unmodified glass and PHEMA-coated surfaces promoted platelet adsorption, leading to activation and formation of pseudopodia. In contrast, a surface coated with PMCP and PHEMA-*g*-PMCP effectively inhibited platelet adhesion, significantly reducing the risk of subsequent thrombus formation. These findings suggest that branched structures combining the properties of PHEMA and zwitterionic polymers are highly effective in minimizing unwanted interactions between surfaces and biological entities, making them promising candidates for biomedical applications.^68^

The application of hydrogel coatings is another effective approach to achieving antifouling surfaces. These cross-linked structures demonstrate high chemical and structural stability, enhancing the resistance of the modified material to adsorption of contaminants, corrosion, or mechanical damage. Utilizing SI-ATRP, Liu et al. developed a hydrogel coating based on *N*-acrylamide glycine (NAG) and *N*-acrylamide glycinamide (NAGA) on a glass surface (Table 2, entry 3).^70^ Notably, the surface modification process did not require an external cross-linker such as methylenebis­(acrylamide) (MBA). This was due to the ability of PNAGA amide groups to form strong hydrogen bonds, inducing hydrogel formation. A critical step in the study was the neutralization of NAG carboxyl groups using NaHCO_3_ before reaction. This was necessary because acidic monomers cannot be polymerized by ATRP techniques, as they irreversibly deactivate catalytic complexes by protonating the ligands. Polymerization was then carried out at different monomer mass ratios (NAGA:NAG = 1:1, 2:1, 5:1, and 19:1), resulting in the formation of four distinct coatings with various thicknesses: 1.31, 1.22, 0.69, and 0.39 μm, respectively. The wettability of the obtained materials was confirmed by water contact angle (WCA) measurements. The highest hydrophilicity was observed in coatings with NAGA:NAG ratios of 1:1 and 2:1 (WCA = 8° vs 30° for bare glass). A highly hydrophilic material should exhibit strong resistance to the adhesion of biological entities. To confirm the antifouling nature of the hydrogels, glass samples were incubated with fluorescent-labeled BSA for 16 h. The most significant decrease in protein adsorption concerning the unmodified surface was observed for the coating with NAGA/NAG ratio of 1:1. This suggests that the hydrophilicity and antifouling effectiveness of the coatings increase with a higher NAG fraction in the hydrogel structure. For transparent materials such as glass, it has been proven that the use of highly hydrophilic films can also reduce the scattering of incident light rays. This occurs because hydrogels prevent the aggregation of condensed water vapor into tensed droplets. Instead, an ultrathin and transparent liquid layer forms on the surface.^71^ To evaluate the antifogging properties of the hydrogels, unmodified and coated glass samples were exposed to a high-humidity environment (RH > 85%) at room temperature. After 30 s, significant fogging was observed on the bare glass, while the hydrogel-modified materials demonstrated much higher transparency. Similar to the antifouling tests, the coating with the highest NAG content exhibited the best antifogging properties (Figure 2c). In addition, both studies demonstrated the remarkable durability of the coatings. After two months of storage, the materials retained their antifouling and antifogging effect. Furthermore, NAGA/NAG hydrogels showed resistance to elevated temperatures (60 °C)—heating for 10 days did not affect the WCA value. Thus, these findings suggest that NAGA/NAG-based coatings have significant potential for use in the development of functional biomaterials.^70^

Materials based on sulfobetaine and carboxybetaine represent another class of coatings willingly used in preventing nonspecific protein adsorption. These zwitterionic compounds are well-known for their biomimetic nature. Sulfobetaine exhibits a structure similar to taurine, which is abundant in animals,^72^ while carboxybetaine resembles glycine betaine, a key compound in osmoregulation in living organisms.^73^ In addition, materials containing both compounds are easy to prepare and show significant stability, a crucial factor for biomedical applications. Zhang et al. polymerized sulfobetaine methacrylate (SBMA) and carboxybetaine methacrylate (CBMA) on a glass surface using SI-ATRP (Table 2, entry 4).^74^ The resulting polymer layers had an estimated thickness of 10–15 nm. For comparison, a random PSBMA-based copolymer was synthesized using free radical polymerization. An enzyme-linked immunosorbent assay (ELISA) revealed minimal fibrinogen adsorption on the PSBMA- and PCBMA-modified glass, showing the level of protein resistance comparable to that of traditionally used PEG-based coatings (Figure 2d). Interestingly, the random PSBMA-based copolymer exhibited much higher fibrinogen adsorption, providing strong evidence that polymers synthesized via ATRP offer superior properties compared to structures obtained by free radical polymerization. Similar trends were noted in studies of bovine aortic endothelial cells (BAECs) adhesion. After 24 h of incubation, BAECs formed a confluent layer on the bare glass surface, whereas no significant adhesion was detected on the PSBMA and PCBMA-modified material. Due to their nontoxicity and excellent resistance to protein and mammalian cells adhesion, these coatings can be successfully used in functionalizing the materials with biomedical applications.^74^

An intriguing approach to modifying glass surfaces involves grafting poly­(ionic liquids) (PILs) onto its surface. PILs affect a wide range of material properties, including hydrophobicity, surface friction and corrosion resistance. Their antifouling and antibacterial effects have also been widely reported in the literature.^75^ He et al. combined ATRP and click chemistry to graft imidazolium-based PILs onto a glass surface.^76^ As an ionic liquid monomer, they used 1-(4-vinylbenzyl)-3-butylimidazolium bis­(trifluoromethylsulfonyl)­imide] (VBBI^+^Tf_2_N^–^), synthesizing both homopolymers and copolymers with *O*-fluorescein methacrylate (FMA) (Table 2, entry 5). The *grafting onto* technique was utilized, which, although it results in lower uniformity and reduced brush grafting density. However, it requires milder conditions than the “*grafting from*” technique. In the paper, thermal azide–alkyne cycloaddition and photoinitiated thiol–ene radical reactions provided successful PILs attachment to the glass surface. This process required the prior functionalization of both the glass surface and polymer chains with “clickable” functional groups. The glass surface was modified with alkene and alkyne moieties, whereas PILs contained thiol (PIL-SH) and azide (PIL-N_3_) groups. Obtaining these chains was possible using specific ATRP initiators. Nevertheless, it is crucial to protect the thiol group before polymerization to prevent unwanted side reactions. The resulting coatings featured higher WCA values compared to unmodified glass, attributed to the hydrophobic nature of the styrenic imidazolium moieties. However, the PILs demonstrated ion-exchange capabilities, allowing their surface properties to be tuned. For example, an anion-exchanged reaction with LiCl replaced the hydrophobic Tf_2_N^–^ ions with highly hydrophilic Cl^–^ ions, leading to increased water absorption and a marked decrease in WCA values. This demonstrates that PIL coatings can be tailored for specific applications by incorporating different counterions into the polymer structure. The combinations of click chemistry and ATRP thus appear to be a promising tool for glass modification, especially for obtaining bioactive, stimuli-responsive, and antifouling surfaces.^76^

Currently, smart polymers are gaining increasing significance in the modification of various types of surfaces due to their ability to easily and reversibly change their properties in response to external factors. Among these, poly­(2-(dimethylamino)­ethyl methacrylate) (PDMAEMA) attracts particular attention due to its simultaneous pH- and thermoresponsive characteristics. The pH sensitivity of this polymer arises from the protonation of amino groups, which enables them to accept H^+^ cations. This protonation introduces positive charges to the polymer’s side chains, leading to electrostatic repulsion between them. This results in a reversible change in the conformation of macromolecules from the globular (collapsed) to the expanded form.^77^ Additionally, the tertiary amino groups in PDMAEMA can be easily quaternized using a wide range of compounds, imparting the material with antibacterial properties. Our group recently introduced a glass surface modification strategy by grafting PDMAEMA brushes using SI-ARGET ATRP (Table 2, entry 6).^53^ This modification was carried out at both the milliliter and microliter scales in a fully aqueous environment. The obtained polymer brushes were then subjected to quaternization with bromoethane. To verify the pH-dependent character of neutral and quaternized (QPDMAEMA) chains, WCA measurements were performed. It has been observed that a decrease in pH below the p*K*_b_ value of PDMAEMA provides a more hydrophilic surface. This occurs because protonated PDMAEMA behaves as a quaternary ammonium ion, enhancing its interaction with water molecules. Conversely, raising the pH above the p*K*_b_ value results in deprotonation of the amino groups, making the surface more hydrophobic due to the transition of PDMAEMA into its free amine form. A similar correlation was observed for QPDMAEMA; nevertheless, the quaternized polymer (as a strong electrolyte) exhibited reduced sensitivity to pH variations compared to its neutral counterpart. Finally, the antibacterial properties of QPDMAEMA-modified glass were investigated against and . Glass slides were incubated with bacterial suspensions for 24 h at 37 °C. To comprehensively assess the bactericidal activity of the obtained materials, several quantitative indicators were determined based on the number of recovered cells after incubation (Table 1).

**Table 1:** Antibacterial Activity of QPDMAEMA-Modified Glass against and^53^

Strain	Survival fraction (SF)	Log reduction (LR) [CFU/mL]	Reduction (PR) [%]
	0.014 ± 0.006	1.86	98.61 ± 0.212
	0.007 ± 0.0003	2.15	99.29 ± 0.07

The antibacterial properties of QPDMAEMA-modified glass have been demonstrated against both bacterial strains, nevertheless, the effect is stronger against the . Antimicrobial activity is usually specified for agents showing an LR value greater than 2, which was not achieved for . However, a significant reduction in the number of living cells of that bacterial strain after 24 h of incubation, i.e., 98.61%, suggests the potential of the coating to reduce glass surface colonization by . In addition to antibacterial evaluation, the protein adsorption of the modified materials was assessed using BSA labeled with Alexa Fluor 488. In contrast to previously reported studies, both QPDMAEMA- and PDMAEMA-modified glass exhibited significantly higher protein adsorption compared to unmodified glass or glass with an immobilized ATRP initiator. As explained earlier, the transition of PDMAEMA side chains to the free amine form within a certain pH range increases the hydrophobicity of the surface, which promotes the adhesion of BSA molecules. This phenomenon could potentially facilitate the growth of other types of cells on the surface (e.g., mammalian cells) by creating a provisional matrix. It is particularly important in regenerative medicine and tissue engineering, which is described in detail later in this article.^53^

Glass modification using RDRP techniques can also be achieved through physical deposition of presynthesized polymer layers onto the material’s surface. Zhu et al. utilized RAFT to prepare homopolymers and random copolymers of 4-vinylpyridine (4VP) and poly­(2-methyl-2-oxazoline) methacrylate macromonomer (PMOXA-MA) (Table 2, entry 7).^78^ PMOXA is a peptidomimetic polymer with high stability and hydration capacity.^79^ Typically, it is synthesized by cationic polymerization of 2-methyl-2-oxazoline, making its binding to the surface a distinct problem. In the reported paper, spin-coating of polymer solutions onto the glass surface was performed, followed by UV irradiation (Figure 2e). The final step is crucial, as it generates active radical centers on 4VP, enabling polymer chain coupling and facilitating the formation of cross-linked structures. These irradiated layers tend to adhere more tightly to the surface due to the reinforced van der Waals interactions. In contrast, nonirradiated coatings can be easily removed from the surface (e.g., by sonication), meaning they do not enable permanent surface modification. The resulting materials were then tested for protein and cell adhesion using fluorescein-labeled BSA, human platelets, and human umbilical vein endothelial cells (HUVECs). The findings revealed that BSA adsorption was significantly reduced on glass coated with irradiated PMOXA-*r*-4VP. Nevertheless, increasing the proportion of 4VP in the copolymer promoted BSA adhesion due to the higher hydrophobicity of the polymer layer. Consequently, P4VP homopolymer-modified glass exhibited high protein adsorption, comparable to the bare material. Similarly, platelet adhesion to the glass surface was reduced when coated with PMOXA and PMOXA-*r*-4VP, whereas P4VP immobilization promoted strong interactions between cells and the material. A similar trend was observed for HUVECs adhesion studies. Therefore, selecting the appropriate initial monomer ratio is essential. The best antiadhesive performance was observed for coatings with [PMOXA]:[4VP] ratios of 1:1 or 1:2. A 3-fold excess of 4VP over PMOXA led to a coating that was not resistant to protein and cell adhesion due to excessive hydrophobicity of the copolymer. Interestingly, a reduction in cell adsorption was also observed on glass modified with nonirradiated PMOXA, likely due to the polymer’s physisorption on the surface. Importantly, the obtained coatings featured high stability over time and demonstrated no cytotoxicity toward HUVECs, highlighting their potential for use in biomaterials development.^78^

**Table 2:** Antiadhesive and Antibacterial Materials Obtained by Glass Modification Using RDRP Techniques

No.	Material	Technique	Monomer	Polymerization conditions	Reference
1	Glass	SI-ATRP	METAC SPMA HEMA	Initiator: chlorosilane 3- (trichlorosilyl)propyl 2-bromo-2-methylpropanoate	^67^
				Catalyst: CuBr/2,2’-bipyridine	
				Argon atmosphere	
				Solvent: H_2_O/isopropanol (synthesis of PMETAC), H_2_O/MeOH (synthesis of PSPMA and PHEMA)	
2	Glass	SI-ATRP	MCP HEMA	Initiator: α-bromoisobutyryl bromide	^68^
				Catalyst: CuBr/bipyridine	
				Nitrogen atmosphere	
				Solvent: H_2_O/MeOH	
				Temperature: 25 °C	
3	Glass	SI-ATRP	NAGA NAG	Initiator: α-bromoisobutyryl bromide	^70^
				Catalyst: CuBr/CuBr_2_/PMDETA	
				Nitrogen atmosphere	
				Solvent: H_2_O	
				Temperature: 25 °C	
4	Glass	SI-ATRP	SBMA CBMA	Initiator: 2-bromo-2-methyl-*N*-3-[(triethoxysilyl)propyl]propenamide	^74^
				Catalyst: CuBr/2,2’-bipyridine	
				Nitrogen atmosphere	
				Solvent: H_2_O/MeOH	
5	Glass Silicon	ATRP Click-chemistry	VBBI^+^ Tf_2_N^–^ FMA	Initiator: 3- azidopropyl 2-bromo-2-methylpropanoate, 3-(acetylthio)propyl 2-bromo-2-methylpropanoate	^76^
				Catalyst: CuBr/CuBr_2_/PMDETA	
				Nitrogen atmosphere	
				Solvent: butyronitrile	
				Temperature: 90 °C	
6	Glass	SI-ARGET ATRP	DMAEMA	Initiator: α-bromoisobutyryl bromide, ethyl α-bromoisobutyrate	^53^
				Catalyst: CuBr_2_/TPMA	
				Air atmosphere	
				Solvent: H_2_O	
				Reducing agent: glucose, fructose	
				Temperature: RT	
7	Glass Silicon Gold PDMS	SI-RAFT	PMOXA-MA 4VP	Initiator: 2, 2’-azobis(isobutyronitrile)	^78^
				Chain transfer agent: S-1-dodecyl-S’-(α,α’-dimethyl-α’’-acetic acid) trithiocarbonate	
				Vacuum atmosphere	
				Solvent: *tert*-butanol	
				Temperature: 80 °C	

## 4 Intelligent Surfaces Responsive to Changes in External Temperature

Stimuli-responsive polymers are a class of materials capable of altering their physicochemical properties in response to external triggers such as temperature,^80^ pH,^81^ light,^82^ glutathione concentration,^83^ and others. Exposure to these stimuli can induce structural changes in the macromolecules, such as bond cleavage or conformational shifts, alter their solubility, or even trigger the generation of reactive oxygen species (ROS). These properties are especially valuable in the design of smart drug delivery systems for cancer therapy or diagnostics.^84^ Among the various stimuli-responsive systems, temperature-sensitive polymers have recently garnered significant attention. This is largely due to their potential for controlled drug release triggered by localized increases in body temperature. A characteristic feature of thermoresponsive polymers is their ability to undergo reversible phase transitions in response to temperature changes. Polymers exhibiting a lower critical solution temperature (LCST) in aqueous environments are soluble below the LCST due to the presence of numerous hydrogen bonds between the polymer chains and surrounding water molecules. Once the temperature exceeds the LCST, these hydrogen bonds are disrupted, leading to increased intra- and intermolecular hydrophobic interactions and a sharp decline in solubility. As a result, thermoresponsive polymers can exist in two distinct thermodynamic states: below the LCST, they form soluble coil-like structures, whereas heating up over the LCST, they collapse into insoluble globules.^80^

Polymers that exhibit LCST primarily include poly­(*N*-substituted acrylamide)­s and ethylene glycol-based polymers. Among them, poly­(*N*-isopropylacrylamide) (PNIPAM) attracts particular attention due to its LCST value of approximately 32 °C, which lies between room temperature and human physiological temperature. This makes NIPAM highly suitable for the synthesis of various copolymers with a precisely defined LCST value, depending on their intended biomedical applications. PNIPAM-based thermoresponsive micelles,^85^ hydrogels,^86^ and polymer brushes^87^ have all been reported. The mechanism of temperature-induced conformational changes in polymer chains grafted from a surface is analogous to that of macromolecules in solution. Below the LCST, polymer brushes are in an extended, hydrated state and exhibit hydrophilic properties. When the temperature exceeds the LCST, the chains collapse into a compact form, rendering the surface more hydrophobic.^80^ Such an easily achievable change in the properties of a given surface provides it with a spectrum of new applications, of which the most commonly used is the ability to manipulate different types of cells.

Extensive work on the use of PNIPAM for glass surface modification via RDRP techniques has been carried out by Prof. Kenichi Nagase’s research team. In one of their foundational studies, they investigated the effects of PNIPAM brush length and grafting density on temperature-dependent fibronectin (FN) adsorption and the adhesion of bovine carotid artery endothelial cells (EC) (Table 3, entry 1).^88^ Prior to polymerization, the glass surface was functionalized with ATRP initiator—2-(*m*/*p*-chloromethylphenyl)­ethyltrimethoxysilane) and phenethyltrimethoxysilane in varying ratios to achieve different grafting densities. Their findings showed that shorter PNIPAM chains and lower grafting densities promoted fibronectin adsorption to the surface at 37 °C. This was attributed to increased exposure of hydrophobic phenethyl groups on the glass surface. In contrast, surfaces with longer and denser PNIPAM brushes exhibited significantly reduced FN adsorption, likely due to decreased availability of hydrophobic sites. Lowering the temperature of the system to 20 °C, below the LCST of PNIPAM, also led to a significant diminution of protein adsorption due to the hydration of the polymer layer. Similarly, EC adhesion was enhanced on surfaces with low PNIPAM grafting density at 37 °C, especially when the brush lengths were short. Longer polymer chains limited hydrophobic interactions, thereby reducing cell adhesion. Cooling to 20 °C enabled efficient cell detachment from surfaces with moderate to high PNIPAM grafting densities. However, surfaces with low grafting density showed poor cell release, suggesting that hydration of the polymer chains was insufficient to overcome the strong hydrophobic interactions between the cells and the exposed phenethyl groups. Therefore, it should be noted that the thermoregulation of antifouling properties and the cell manipulation ability of PNIPAM-modified surfaces strictly depend on the optimization of chain length and their grafting density.^88^

**Table 3:** Thermoresponsive Surfaces Obtained by Glass Modification Using RDRP Techniques

No.	Technique	Monomer	Polymerization conditions	Reference
1	SI-ATRP	NIPAM	Initiator: 2-(m/*p*-chloromethylphenyl)ethyltrimethoxysilane	^88^
			Catalyst: CuCl/CuCl_2_/Me_6_TREN	
			Nitrogen atmosphere	
			Solvent: isopropanol	
			Temperature: 25 °C	
2	SI-ATRP	NIPAM	Initiator: 2-(m/*p*-chloromethylphenyl)ethyltrimethoxysilane, α-chloro-*p*-xylene	^89^
			Catalyst: CuCl/Me_6_TREN	
			Argon atmosphere	
			Solvent: isopropanol	
			Temperature: 25 °C	
3	SI-ATRP	NIPAM *n*BMA	Initiator: 2- (m/*p*-chloromethylphenyl)ethyltrimethoxysilane, α-chloro-*p*-xylene	^90^
			Catalyst: CuCl/Me_6_TREN	
			Argon atmosphere	
			Solvent: isopropanol	
			Temperature: 25 °C	
4	SI-ARGET ATRP	NIPAM DMAPAM	Initiator: [(chloromethyl)phenylethyl] trimethoxysilane, α-chloro-*p*-xylene	^91^
			Catalyst: CuCl_2_/Me_6_TREN	
			Reducing agent: ascorbic acid	
			Solvent: H_2_O/isopropanol	
			Temperature: 25 °C	
5	SI-RAFT	NIPAM BzMA	Initiator: 4,4’-azobis(4-cyanovaleric acid)	^92^
			Chain transfer agent: 4-cyanopentanoic acid dithiobenzoate	
			Nitrogen atmosphere	
			Solvent: 1,4-dioxane	
			Temperature: 70 °C	
6	SI-RAFT	NIPAM	Initiator: 4,4′-azobis-4-cyanovaleric acid	^93^
			Chain transfer agent: 4-cyanopentanoic acid dithiobenzoate	
			Nitrogen atmosphere	
			Solvent: 1,4-dioxane	
			Temperature: 70 °C	
7	SI-ARGET ATRP	P4VP POEGMA_188_	Initiator: α-bromoisobutyryl bromide	^94^
			Catalyst: CuBr_2_/2,2’-bipyridine	
			Nitrogen atmosphere	
			Solvent: MeOH/H_2_O	
			Reducing agent: sodium L-ascorbate	
			Temperature: RT	
8	SI-ARGET ATRP	POEGMA_188_	Initiator: α-bromoisobutyryl bromide	^98^
			Catalyst: CuBr_2_/2,2’-bipyridine	
			Nitrogen atmosphere	
			Solvent: MeOH/H_2_O	
			Reducing agent: sodium L-ascorbate	
			Temperature: RT	
9	SI-ARGET ATRP	*n*BMA *n*BA	Initiator: α-bromoisobutyryl bromide	^99^
			Catalyst: CuBr_2_/2,2’-bipyridine	
			Nitrogen atmosphere	
			Solvent: MeOH/H_2_O	
			Reducing agent: sodium L-ascorbate	
			Temperature: RT	

Another study investigated the effect of PNIPAM polymer brush length on the thermally regulated adhesion of four types of human cells on a glass surface (Table 3, entry 2).^89^ For this purpose, human umbilical vein endothelial cells (HUVECs), normal human dermal fibroblasts (NHDFs), human aortic smooth muscle cells (SMCs), and human skeletal muscle myoblast cells (HSMMs) were used. The cells were initially seeded onto the surface at 37 °C, allowing them to adhere, and then tested for their ability to detach at 20 °C. At the elevated temperature, all cell types adhered strongly to the glass surfaces modified with short polymer brushes (*M*_n_ = 8200). However, cooling the system below LCST did not lead to cell detachment due to the low mobility of short PNIPAM chains and insufficient hydration of the polymer layer. The medium-length polymer brushes (*M*_n_ = 12800) promoted slightly less efficient cell adhesion to the surface at 37 °C but exhibited sufficient hydration upon cooling, which facilitated detachment of all four cell types at varying rates. Among them, HUVECs exhibited the highest detachment efficiency. To demonstrate the potential application in cell separation, the researchers used this material to isolate a mixture of green fluorescent protein-expressing HUVECs (GFP-HUVECs) and HSMMs. Most of the GFP-HUVECs detached from the surface immediately after cooling the system to 20 °C, while HSMMs remained on the surface longer and detached gradually with continued incubation. This enabled efficient temperature-controlled separation of two cell types. In contrast, long PNIPAM brushes (*M*_n_ = 15600) exhibited high mobility and hydrophilicity, which prevented effective cell adhesion. Consequently, surfaces modified with long PNIPAM chains were unsuitable for cell culture or manipulation. These findings highlight the importance of optimizing PNIPAM brush length to develop functional materials for thermoresponsive cell separation. Glass surface modified with medium-length PNIPAM brushes is expected to be useful as field flow fractionation (FFF) and cell separation chromatography matrices.^89^

Thermoregulated adhesion of the same set of cells has also been studied on glass surfaces modified with random copolymers of NIPAM and *n*-butyl methacrylate (*n*BMA) (Table 3, entry 3).^90^ As the *n*BMA content in the polymer brushes increased, the LCST value decreased compared to that of PNIPAM homopolymer. This effect resulted directly from the enhanced hydrophobicity of the copolymer, which promoted dehydration at lower temperatures. Increasing the hydrophobicity of the polymer brushes additionally facilitated stronger adhesion of all cell types due to enhanced surface-cell hydrophobic interactions. Glass surface modified with polymer brushes containing a 5 mol % of BMA feed composition exhibited cell adhesion at 37 °C comparable to that observed with tissue culture polystyrene (TCPS). Each cell type exhibited a different effective detachment temperature. HUVECs detached efficiently at 20 °C, while NHDFs required lowering the temperature to 10 °C due to stronger interactions with the surface (Figure 3a). SMCs and HSMMs demonstrated similar detachment profiles at both 20 and 10 °C, indicating weak temperature sensitivity. Therefore, P­(NIPAM-*co*-*n*BMA)-modified glass can be utilized to efficiently separate HUVECs and NHDFs mixtures through stepwise temperature changes. This method = does not require additional surface modification of the cells, preserving their biological activity and making them suitable for tissue engineering applications.^90^

**Figure 3:**
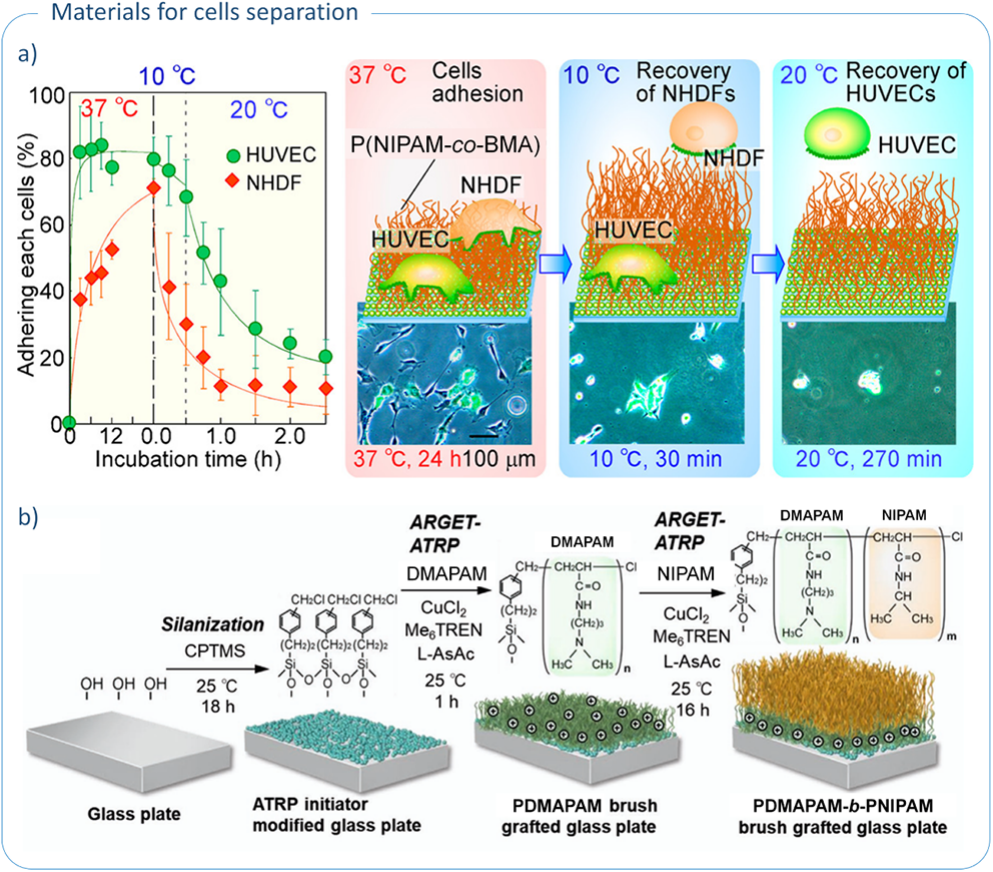
Functionalized glass as a tool for cell separation: a) thermoregulated cell separation on P­(NIPAM-*co*-BMA)-modified glass surface; reproduced with permission from ref. 90. Copyright 2013, American Chemical Society; b) general scheme of glass modification by PDMAPAM-*b*-PNIPAM block copolymers; reproduced with permission from ref. 91. Copyright 2020, Wiley-VCH GmbH.

In another study, PNIPAM block copolymers containing *N*,*N*-dimethylaminopropylacrylamide (DMAPAM) were grafted from glass surface via SI-ARGET ATRP to produce materials with selective cell manipulation capabilities (Table 3, entry 4).^91^ The resulting polymer brushes were composed of an inner cationic PDMAPAM block and an outer thermoresponsive PNIPAM block (Figure 3b). Three types of polymer chains were obtained, differing in the length of the thermosensitive segment. Adhesion tests were performed using NHDFs, RAW264.7 cells (macrophages), and umbilical cord-derived mesenchymal stem cells (UCMSCs) as target cells. At 37 °C, all cell types adhered tightly to a highly cationic copolymer containing a short PNIPAM block. Nevertheless, lowering the temperature to 20 °C did not trigger detachment, due to the insufficient hydrophilicity of the polymer layer. Increasing the length of the PNIPAM segment reduced surface interactions and promoted detachment of all cell types upon cooling, eliminating selectivity. Selective, thermoregulated cell adhesion was only achieved for glass surfaces modified with polymer brushes containing a moderate-length PNIPAM block. At 20 °C, UCMSCs detached almost completely, while NHDFs and RAW264.7 remained attached, which enabled their selective separation. The degree of cell adhesion was influenced by zeta potential of each cell type. For instance, NHDFs exhibited a more negative zeta potential than UCMSC (−13,27 mV and −9,04 mV, respectively), resulting in stronger electrostatic interactions with the cationic PDMAPAM segment and effectively preventing their detachment. To validate the material’s ability to selectively isolate UCMSC, a 1:1:1 mixture of all three cell types was incubated on the surface. After cooling to 20 °C, UCMSCs were selectively recovered with about 70% efficiency, while NHDFs and macrophages remained attached. Importantly, the recovered UCMSC retained their morphology, metabolic activity, and proliferative capacity, confirming the suitability of this thermoresponsive cationic copolymer-modified glass for stem cell purification in therapeutic applications.^91^

The effect of a second block on the properties of PNIPAM-based thermoresponsive polymer brushes was also investigated by Matsuzaka et al. (Table 3, entry 5).^92^ In this study, the inner segment was composed of highly hydrophobic poly­(benzyl methacrylate) (PBzMA). Bovine carotid artery endothelial cells (BAECs) adhesion tests were conducted on glass surfaces modified with three types of polymer brushes. As expected, PNIPAM homopolymer brushes promoted cell adhesion at 37 °C and further cell proliferation (Figure 4a). The presence of a short block of PBzMA in the copolymer brushes did not significantly affect the ability of the cells to adhere. However, when the temperature was lowered to 20 °C, cell detachment occurred more rapidly compared to the surface modified with PNIPAM homopolymers. The detachment rate further increased with copolymers containing a longer PBzMA block. Nevertheless, it should be noted that brushes with long PBzMA blocks inhibited cell adhesion due to their excessive hydrophobicity, preventing the formation of a complete cell sheet. In contrast, the other two surface types effectively supported the formation of a confluent BAECs monolayer during incubation at 37 °C. Importantly, the cell sheets detached much more rapidly from the PBzMA-*b*-PNIPAM brush-modified surface. This can be explained by the repulsive effect of the overly hydrophobic PBzMA block on the cell-surface interaction. Reducing the time required to harvest intact cell sheets from culture surfaces is a highly desirable phenomenon in tissue engineering (Figure 4b). Therefore, glass surfaces modified with PBzMA-*b*-PNIPAM brushes represent a promising approach for the reconstruction of damaged organs and tissues.^92^

**Figure 4:**
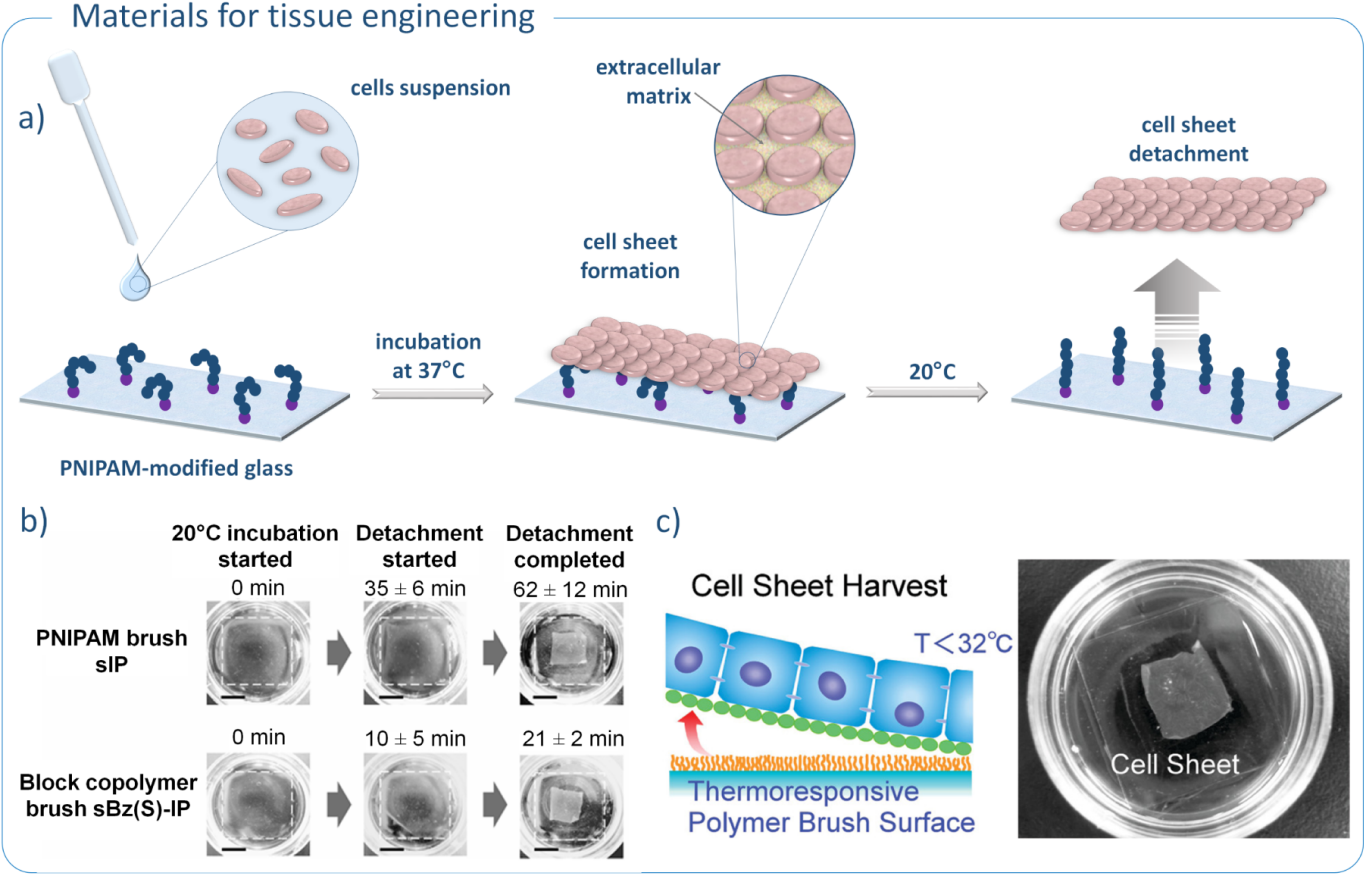
Polymer-modified glass for tissue engineering: a) general mechanism of cell sheets formation on PNIPAM-modified glass surface; b) cell sheets development on PNIPAM-modified glass surface; reproduced with permission from ref. 92. Copyright 2012, Taylor & Francis c) cell sheet detachment from thermoresponsive PNIPAM layer; reproduced with permission from ref. 93. Copyright 2010, American Chemical Society.

The possibility of culturing BAECs cell sheets on PNIPAM-modified glass surface was also investigated by Takahashi et al. (Table 3, entry 6).^93^ The ability of cells to adhere to the surface, form a confluent monolayer, and detach as intact cell sheets is dependent on both the length and grafting density of PNIPAM chains. Shorter polymer brushes facilitated faster cell adhesion at 37 °C, while longer PNIPAM chains increased surface hydrophilicity at 20 °C, promoting more efficient cell detachment. An optimal balance between adhesion and detachment was achieved, as expected, with PNIPAM brushes of medium length. Low grafting density of the polymer chains provided stronger BAECs interactions with the surface, whereas high grafting density enabled faster changes in hydrophilicity in response to temperature shifts, leading to quicker cell detachment. As a result, rapid and efficient harvesting of intact BAEC sheets was accomplished by modifying the glass surface with PNIPAM brushes of medium length and high grafting density. Importantly, cell sheets were detached along with the intact extracellular matrix (ECM), a key factor for their application in regenerative medicine (Figure 4c). Moreover, by fine-tuning the length and grafting density of PNIPAM chains, the surface could be tailored to suit different cell types. For instance, surfaces with lower grafting densities were more suitable for culturing cells with lower adhesiveness, such as hepatocytes. Thus, glass modified with thermosensitive PNIPAM brushes represents a versatile and smart platform for culturing a wide range of cell types, regardless of their proliferation properties or ATP-dependent metabolism.^93^

Modification of the glass surface with thermoresponsive polymer brushes can also impart temperature-dependent antibacterial properties. Raczkowska et al. developed intelligent carriers for silver nanoparticles (AgNPs) on glass using SI-ARGET ATRP (Table 3, entry 7).^94^ Two types of surfaces were prepared by grafting poly­(4-vinylpyridine) (P4VP) and poly­(di­(ethylene glycol)­methyl ether methacrylate) (POEGMA_188_) chains. Both polymers are capable of undergoing reversible phase transitions at 10–15 °C and 18–26 °C, respectively.^95,96^ Silver ions (Ag^+^) were deposited onto the polymer layers and subsequently reduced to AgNPs using sodium borohydride (NaBH_4_). The antibacterial properties of the resulting materials were then evaluated against and . Both bacterial strains were incubated on the modified surfaces at two different temperatures. At 4 °C, the bacteria demonstrated a high survival rate, while incubation at 37 °C led to significant bacterial death. The temperature-dependent biocidal effect can be attributed to the controlled release of Ag^+^ ions when the system is heated above the LCST. In addition, the collapse of the polymer brushes above the LCST allows better contact of silver nanoparticles with bacterial membranes, leading to their destabilization and cell death. Notably, the P4VP coating also displayed partial antimicrobial activity against in the absence of AgNPs, likely due to the electrostatic interaction between the polymer’s charged surface and the bacterial cell membrane. The developed approach holds promise for applications in environments where elevated temperatures promote bacterial growth, such as in the food industry.^94^

However, the widespread use of AgNPs as an antibacterial agent is limited due to their cytotoxicity and genotoxicity. In addition to their tendency to induce oxidative stress, DNA damage, or apoptosis, AgNPs display the ability to accumulate in various organs, such as the liver, kidneys, and brain.^97^ Nevertheless, AgNPs appear to be an attractive anticancer agent, as their cytotoxicity against tumor cells is considerably higher compared to normal cells. To investigate this further, the same research group evaluated the cytotoxicity and antibacterial effect of AgNPs immobilized on the surface of POEGMA_188_-modified glass (Table 3, entry 8).^98^ As expected, the nanocomposite coatings demonstrated strong antibacterial activity against and , with efficacy increasing alongside the AgNPs content. When spontaneously transformed human keratinocytes from histologically normal skin (HaCaT) were incubated on the coated surface, a confluent monolayer of cells formed within 72 h. On the other hand, far fewer WM35 cancerous melanoma cells accumulated on the nanocomposite layer, even after prolonged incubation. These findings suggest, though do not conclusively prove, the potential antitumor effect of immobilized AgNPs. Nevertheless, it should be explicitly noted that the presence of AgNPs in the coating did not affect the proliferative capacity of HaCaT. This is likely due to the prolonged release of silver ions from the polymer layer, preventing the accumulation of cytotoxic concentrations. Consequently, these nanocomposites appear to be safe for contact with normal cells, making them a promising material for applications in the food industry or medical laboratories.^98^

The properties of a surface modified with polymer brushes can also be influenced by the polymer’s ability to reversibly transform from glassy to rubbery state (Table 3, entry 9).^99^ In this study, glass surfaces were functionalized with poly­(*n*-butyl acrylate) (P*n*BA) and poly­(*n*-butyl methacrylate) (P*n*BMA) brushes using SI-ARGET ATRP technique. Despite minor structural differences, these polymers exhibit significantly different glass transition temperatures (*T*_g_): P*n*BA has a *T*_g_ of −55 °C, while P*n*BMA has a *T*_g_ ranging from 13 to 25 °C, making it particularly attractive for biomedical applications. The morphology of the obtained coatings was studied by atomic force microscopy (AFM) across a temperature range of 5 to 35 °C. As the temperature increased, the P*n*BMA coating underwent a visible transformation from a coarse to a smooth surface, the P*n*BA coating showed no noticeable change. This temperature-dependent behavior directly affected the adsorption of proteins. As the temperature increased from 10 to 35 °C, the amount of BSA adsorbed on the P*n*BMA coating doubled, while protein accumulation on the P*n*BA layer was constant and independent of temperature. The glass transition of P*n*BMA also influenced the orientation of adsorbed protein molecules. Below *T*_g_, BSA tended to orient with domains 1 and 2 (rich in amino acids such as tyrosine, histidine, and serine) facing outward. At elevated temperatures (above *T*_g_), the orientation changed, and the domain 3 (rich in valine and threonine) became exposed. A similar thermoregulated orientation change was observed for anti-IgG antibodies. The change in orientation affected the immunological properties of the anti-IgG. For antibodies adsorbed at lower temperatures, the end-on orientation was dominant, where the F­(ab′)_2_ fragment, responsible for specific antigen binding, was more exposed. Exceeding the *T*_g_ value promoted a head-on orientation, in which the Fc fragment was directed outward. As a result, antibodies adsorbed below the *T*_g_ exhibited greater antigen-binding efficiency. The presented concept of thermal regulation of protein orientation on polymer brush-modified surfaces appears to be a promising strategy for obtaining materials with dynamically controlled biological properties.^99^

## 5 Biocompatible Platforms for Biomolecule Immobilization

Coupling synthetic polymers with natural macromolecules such as peptides, proteins, saccharides, or nucleic acids is a promising strategy for designing materials with unique properties. These bioconjugates hold significant value in medicine, nanotechnology, and materials engineering. They are commonly used to stabilize therapeutic proteins or to design intelligent drug and gene delivery systems. Therefore, precise control over the structure of these bioconjugates and a clear understanding of the mechanisms governing polymer–biomolecule interactions are crucial for advancing their applications.^100^

An innovative approach in the development of smart materials involves the coupling of naturally derived molecules with polymer brushes grafted from various surfaces. Tugulu et al. utilized SI-ATRP to graft PHEMA and poly­(poly­(ethylene glycol) methacrylate) (PPEGMA) brushes from a glass surface (Table 4, entry 1).^101^ The resulting polymer chains were subsequently functionalized with short peptide ligands based on the RGD sequence—a key motif within the fibronectin domain responsible for binding to integrin receptors on the cell surface. The modified materials were evaluated for their specific adhesion and proliferation of HUVECs. The RGD-functionalized surface strongly promoted cell adhesion, whereas unmodified brushes and those conjugated with a control peptide did not support cell adhesion. However, the interactions between the material and HUVECs were dependent on the RGD concentration on the surface. High adhesion levels were observed on surfaces with RGD at concentrations ≥ 5.3 pmol/cm^2^, while at low concentrations of the peptide (0.2–1.0 pmol/cm^2^), the cells adhered poorly and displayed the presence of elongated protrusions, suggesting insufficient integrin interactions. The cell response was also influenced by the length of PPEGMA. The longer side chains increased the mobility of the RGD ligand, which weakened their interactions with cell surface receptors. In addition to promoting adhesion, RGD-functionalized polymer brushes supported the formation of a confluent HUVEC monolayer. Notably, the adherent cells exhibited a physiological response when exposed to a shear stress mimicking blood flow, demonstrating the material’s potential for various cardiovascular applications.^101^

**Table 4:** Platforms for Biomolecule Immobilization Obtained by Glass Surface Modification Using ATRP

No.	Material	Technique	Monomer	Polymerization conditions	Reference
1	Glass Silicon wafers	SI-ATRP	HEMA PEGMA	Initiator: 3-(2-bromoisobutyramido)propyl(trimethoxy) silane	^101^
				Catalyst: CuCl/CuBr_2_/2,2’-bipyridine (synthesis of PHEMA and PPEGMA), CuBr/CuBr_2_/2,2’-bipyridine (synthesis of PPEGMA)	
				Nitrogen atmosphere	
				Solvent: H_2_O (synthesis of PHEMA), H_2_O/MeOH (synthesis of PPEGMA)	
				Temperature: RT (synthesis of PHEMA), 60 °C (synthesis of PPEGMA)	
2	Glass	SI-ATRP	HEMA PEGMA	Initiator: 3-(2-bromoisobutyramido)propyl(trimethoxy)silane	^102^
				Catalyst: CuCl/CuBr_2_/2,2’-bipyridine (synthesis of PHEMA), CuBr/CuBr_2_/2,2’-bipyridine (synthesis of PPEGMA)	
				Nitrogen atmosphere	
				Solvent: H_2_O (synthesis of PHEMA), H_2_O/MeOH (synthesis of PPEGMA)	
				Temperature: RT (synthesis of PHEMA), 60 °C (synthesis of PPEGMA)	
3	Glass fiber	SI-ICAR ATRP	GMA	Initiator: α-bromoisobutyryl bromide, ethyl 2-bromo-2-methylpropionate, 2,2’-azobis(isobutyronitrile)	^104^
				Catalyst: CuBr_2_/TPMA	
				Argon atmosphere	
				Solvent: anisole	
				Temperature: 60 °C	

Another paper describes the concept of modifying the glass surface with PHEMA and poly­(oligo­(ethylene glycol) methacrylate) (POEGMA) brushes functionalized with *O* ^6^-benzylguanine derivative (BG)^102^ (Table 4, entry 2). As expected, grafting of polymer chains prevented nonspecific adsorption of proteins on the glass surface. Nevertheless, further functionalization of the coating with BG provided a POEGMA-modified surface with the ability to selectively immobilize AGT (*O* ^6^-alkylguanine-DNA alkyltransferase) fusion proteins (Figure 5a). This selectivity is due to the unique activity of AGT, a DNA repair enzyme that catalyzes guanine dealkylation reactions by transferring an alkyl group to one of its cysteine residues. By introducing BG onto the polymer brush layer, specific binding of AGT-containing proteins was achieved via a chemoselective alkyl group transfer reaction. This selective immobilization method offers several advantages, such as the ability to isolate well-specified proteins from cell lysates without the need for other purification steps. Consequently, glass surfaces modified with BG-functionalized polymer brushes show great potential for use in the development of protein microarrays.^102^

**Figure 5:**
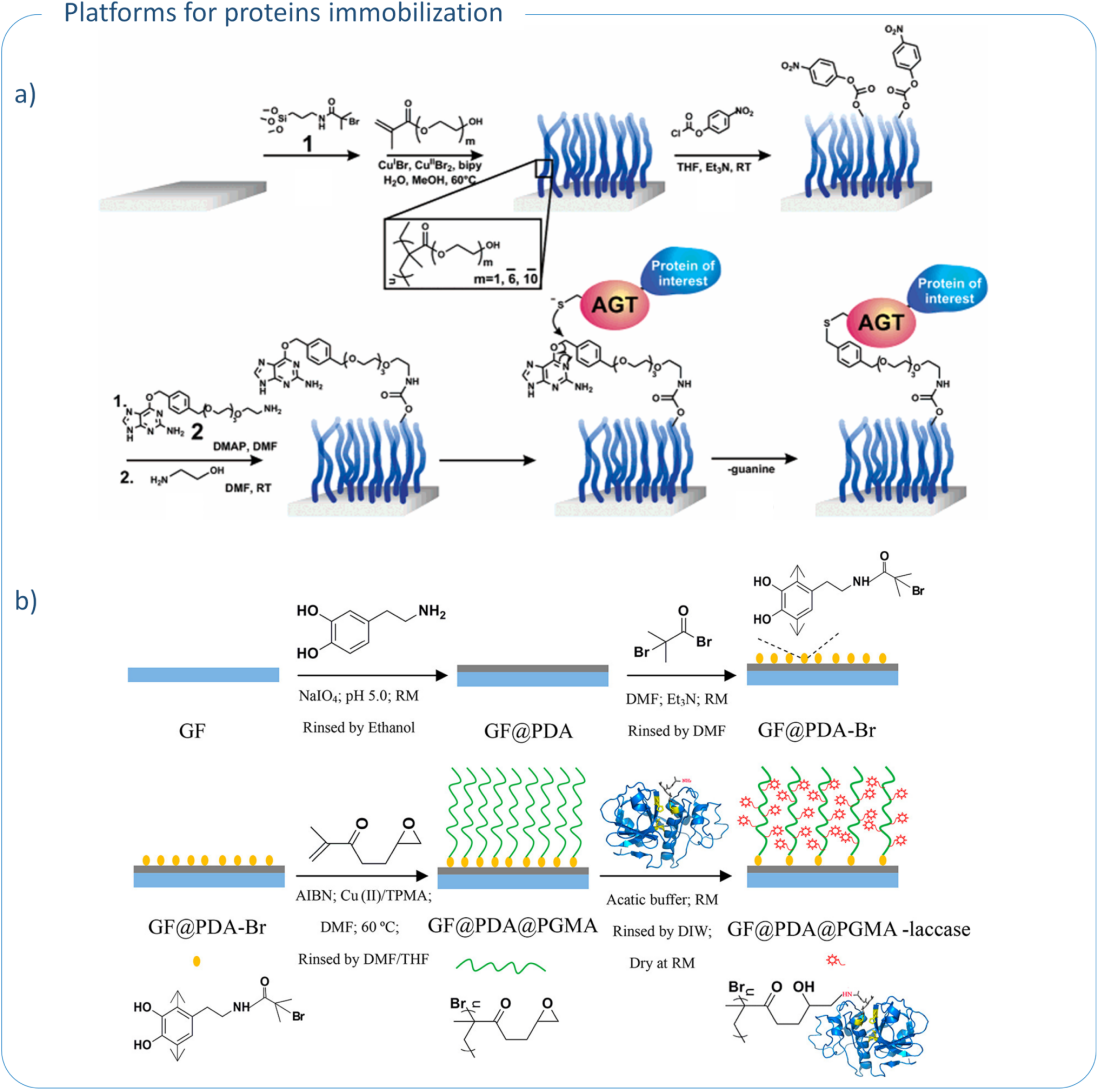
General scheme of glass functionalization for a) AGT fusion proteins immobilization; reproduced with permission from ref. 102. Copyright 2005, American Chemical Society; b) laccase immobilization; reproduced with permission from ref. 104. Copyright 2017, American Chemical Society.

Immobilization of proteins on solid supports is a frequently used strategy to improve the stability and catalytic activity of many enzymes.^103^ Wang et al. developed the concept of immobilizing laccase on glass fiber carrier.^104^ Laccase is an enzyme that catalyzes the oxidation reaction of various phenolic compounds and aromatic amines.^105^ In their study, the surface of glass fibers was first coated with a thin and homogeneous layer of polydopamine (PDA), which facilitated the attachment of ATRP initiator through an amidation. Subsequently, poly­(glycidyl methacrylate) (PGMA) brushes were grafted from the fiber surface using ICAR ATRP technique (Table 4, entry 3). The presence of reactive epoxy groups in PGMA side chains enabled covalent immobilization of laccase through a reaction between the oxirane rings of the polymer and the amino groups of the enzyme (Figure 5b). The catalytic activity of the immobilized laccase was then assessed by its ability to degrade 2,6-dimethoxyphenol (DMP). Compared to free enzyme, the immobilized laccase displayed significantly higher activity and stability, even under mechanical shear conditions. This approach, which combines bioinspired PDA chemistry with the versatility of enzyme immobilization, highlights the potential of PGMA-modified glass fiber composites for applications in industrial biocatalysis.^104^

## 6 Another Potential Application of Polymer-Modified Glass: A Field for Further Development

As previously mentioned, the concept of modifying glass surface with polymer brushes is currently a keenly researched topic. Obtaining antifouling materials, smart stimuli-responsive surfaces, and platforms for immobilizing biological entities appears to be an essential and well-investigated part of modern materials science. However, there remain numerous unexplored opportunities for glass-polymer composites, which may have potential applications yet to be fully explored.

For instance, the modified glass was applied as a biosensor for DNA analysis.^106^ For this purpose, the glass surface was functionalized with either pure oligonucleotide coatings or mixed with PHEMA films (Table 5, Entry 1). The presence of polymer brushes significantly enhanced the hydrophilicity of the surface, facilitating the interaction of the potential sensor with biological solutions. As expected, the PHEMA coating prevented the nonspecific adsorption of contaminants on the surface, leading to a much higher signal-to-background ratio during DNA detection. The surface modification also improved the stability and selectivity of the designed biosensor, enabling more accurate determination of differences in the thermal melting temperatures (*T*_m_) of DNA hybrids. These findings suggest that PHEMA brush-modified glass could serve as a valuable tool for detecting genetic mutations, such as single-nucleotide polymorphism (SNP).^106^

**Table 5:** Glass-Based Hybrid Materials with Another Potential Application

No.	Material	Technique	Monomer	Polymerization conditions	Reference
1	Glass Silicon	SI-ATRP	HEMA	Initiator: bromoisobutyryl *N*-hydroxysuccinimide ester	^106^
				Catalyst: CuCl/CuBr_2_/2,2’-bipyridine	
				Argon atmosphere	
				Solvent: H_2_O	
				Temperature: RT	
2	Glass	SI-saATRP	NIPAM NAPMAM	Initiator: [(chloromethyl)phenylethyl]trimethoxysilane	^107^
				Catalyst: CuCl_2_/Me_6_TREN	
				Air atmosphere	
				Reducing agent: Zn	
				Solvent: DMF/H_2_O	
				Temperature: 25 °C	

In another application, polymer brushes grafted from glass surfaces were used in the design of autonomous nanoactuators. Masuda et al. utilized sacrificial-anode ATRP (saATRP) to graft gradient random copolymers of PNIPAM and poly­(*N*-3-(aminopropyl)­methacrylamide) (PNAPMAM) on the glass surface (Table 5, entry 2).^107^ These polymer chains were then functionalized with ruthenium tris­(2,2′-bipyridine) (Ru­(bpy)_3_), a catalyst for the Belousov–Zhabotinsky (BZ) reaction. A zinc electrode was used to create a polymer brush gradient, with chain length ranging from 34 to 77 nm (Figure 6). The BZ reaction triggered self-oscillating redox changes in the Ru­(bpy)_3_, resulting in cyclic swelling and shrinking of the polymer brushes. This system exhibited unidirectional movement of the chemical wave, propagating from areas with a lower to higher concentrations of Ru­(bpy)_3_. The gradient in polymer chain length played a critical role in controlling the direction of wave propagation, as it directly affected the amount of immobilized catalyst. Reversing the Ru­(bpy)_3_ concentration gradient also reversed the direction of wave motion, underscoring the importance of this factor Notably, changing the parameters of the BZ reaction, such as the concentration of NaBrO_3_ (one of the substrates) or temperature, affected the speed of wave propagation, but did not change its direction. This proved that the polymer brush gradient is the dominant factor controlling the oscillatory process. The use of saATRP to modify glass appears to be an interesting concept in the design of intelligent autonomous mass transport. This biomimetic concept, inspired by ciliary motion, could enable fluid flow through the directional propagation of chemical waves.^107^

**Figure 6:**
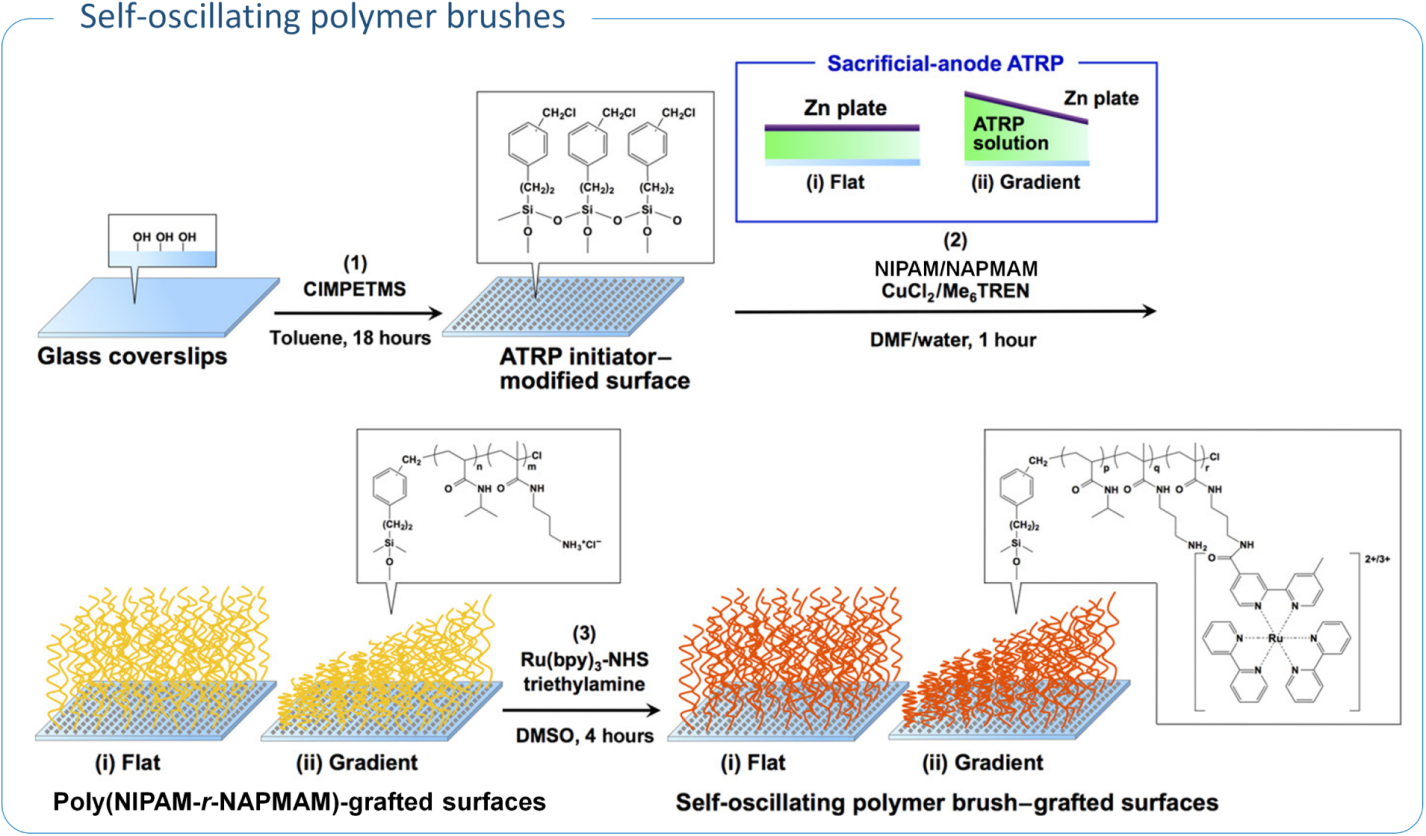
General scheme of glass surface modification with self-oscillating polymer brushes; reproduced with permission from ref. 107. Copyright 2016, The American Association for the Advancement of Science.

## 7 Summary and Future Outlooks

Glass surface modification by RDRP techniques has gained recognition in recent years as a promising concept in obtaining innovative materials. This growing interest is primarily due to the ability to impart many unique properties to the surface, thereby expanding their potential applications in both modern science and industry. The opportunity to graft polymers with diverse chemical characteristics from the glass surface opens up the possibility of achieving even more interesting materials with still undiscovered applications. One particularly promising avenue is the use of polymer-modified glass in the development of modern diagnostic systems. For example, such materials could find application in the manufacturing of sensors for the selective detection of body fluid components or toxins present in food and water. The design of glass-based components for diagnostic systems holds significant promise for improving the monitoring of disease progression and treatment response, ultimately enabling faster and more effective therapeutic outcomes. Glass, as a structural material, also contributes to sustainability due to its superior mechanical durability compared to common plastic alternatives. This enhanced longevity reduces the need for frequent component replacement, thereby minimizing waste and improving the overall reliability and cost-efficiency of diagnostic operations. Polymer-modified glass could also serve as a catalyst carrier in heterogeneous catalysis—an area of great significance in contemporary industrial chemistry.

As previously mentioned, over the decades, glass has been employed in the preparation of therapeutic biomaterials, such as bone implants.^28^ Modification of glass surface by grafting polymer offers the possibility of creating implants with enhanced biocompatibility and the ability to integrate with bone tissue. Moreover, since polymer coatings can act as smart drug carriers, these implants could be functionalized with analgesics or antimicrobial agents, thereby reducing the risk of local inflammations or biomaterial-centered infections. Functionalizing glass surfaces using RDRP techniques offers a highly promising approach for developing advanced materials that enhance patient comfort in the management of chronic injuries and improve therapeutic efficacy across various medical conditions.

It is particularly worthwhile to continue research into the development of smart surfaces capable of manipulating biological material. This is especially relevant given the crucial role tissue engineering plays in regenerative medicine today. Hence, the continuous development of materials that promote the creation of durable cell sheets for therapeutic applications is necessary. However, to fully harness this potential, a deeper understanding of the mechanisms governing cell interactions with polymer coatings is essential. Such knowledge will enable the design of materials with even greater immobilization selectivity, tailored to a broader range of cell types. The proposed strategy holds strong potential for application in next-generation bioseparation technologies, particularly in cell chromatography systems. These methods are increasingly important in the context of rapidly advancing cell-based therapeutic platforms, including those involving stem cells. Additionally, integrating such approaches into the design of advanced diagnostic and analytical devices could significantly enhance the efficiency, specificity, and scalability of cell sorting and analysis procedures, thereby supporting both clinical and research-driven progress in regenerative medicine and molecular diagnostics.

However, the widespread adoption of these concepts in the development of materials for biotechnological and medical applications requires further in-depth investigation. Continued research is essential to establish more cost-effective and environmentally sustainable surface functionalization strategies. Among the most promising avenues are light-mediated RDRP techniques, such as photoATRP, metal-free ATRP, and photoinduced electron/energy transfer (PET) RAFT polymerization. These methods typically use visible or near-infrared light to control polymer chain growth, including from substrate surfaces.^108–112^ This light-driven regulation can significantly reduce—or even eliminate—the need for certain reagents, such as metal-based catalytic complexes used in ATRP, thereby lowering overall process costs. Such simplification is especially advantageous for biomedical applications, where high product purity is critical.

Importantly, none of these light-mediated techniques have yet been applied to the functionalization of glass-based materials, highlighting a valuable opportunity for further exploration. Moreover, incorporating reaction systems that use naturally derived reagents, such as biobased solvents or reducing agents,^37,49,113,114^ represents another promising direction. Like light-mediated polymerizations, these biobased systems can help reduce production costs, improve safety for both operators and end-users, and enhance the commercial viability of the resulting materials.

## Abbreviations

4VP: 4-vinylpirydine
AFM: atomic force microscopy
AgNPs: silver nanoparticles
AGT: *O* ^6^-alkylguanine-DNA alkyltransferase
AIBN: azobis­(isobutyronitrile)
ARGET: activators regenerated by electron transfer
AsAc: ascorbic acid
ATP: adenosine triphosphate
ATRP: atom transfer radical polymerization
BAECs: bovine aortic endothelial cells
BCI: biomaterial-centered infections
BG: *O* ^6^-benzylguanine derivative
BiBB: α-bromoisobutyryl bromide
BMA/*n*BMA: *n*-butyl methacrylate
BSA: bovine serum albumin
BZ: Belousov–Zhabotinsky
CBMA: carboxybetaine methacrylate
CP: choline phosphate
CPTMS/CIMPETMS: [(chloromethyl)­phenylethyl] trimethoxysilane
CTA: chain transfer agent
CuAAC: copper-catalyzed azide–alkyne cycloaddition
DIW: deionized water
DMAP: 4-dimethylaminopyridine
DMAPAM: *N*,*N*-dimethylaminopropylacrylamide
DMF: *N,N*-dimethylformamide
DMP: 2,6-dimethoxyphenol
DNA: DNA
EC: bovine carotid artery endothelial cells
ECM: extracellular matrix
ELISA: enzyme-linked immunosorbent assay
Et_3_N: triethylamine
FMA: *O*-fluorescein methacrylate
FN: fibronectin
GF: glass fiber
GFP-HUVECs: green fluorescent protein expressing human umbilical vein endothelial cells
HaCaT: spontaneously transformed keratinocytes from histologically normal skin
HSMMs: human skeletal muscle myoblast cells
HUVECs: human umbilical vein endothelial cells
ICAR: initiators for continuous activator regeneration
LCST: lower critical solution temperature
LR: log reduction
MBA: methylenebis­(acrylamide)
MCP: 2-(methacryloyloxy)­ethyl choline phosphate
Me_6_TREN: tris­[2-(dimethylamino)­ethyl]­amine
MeOH: methanol
*M*_n_: number-average molar mass
NAG: *N*-acrylamide glycine
NAGA: *N*-acrylamide glycinamide
NHDFs: normal human dermal fibroblasts
NMP: nitroxide mediated polymerization
P4VP: poly­(4-vinylpirydine)
PBA/P*n*BA: poly­(*n*-butyl acrylate)
PBzMA: poly­(benzyl methacrylate)
PDA: polydopamine
PDMAEMA: poly­(2-(dimethylamino)­ethyl methacrylate)
PDMS: polydimethylsiloxane
PEG: poly­(ethylene glycol)
PGMA: poly­(glycidyl methacrylate)
PHEMA: poly­(2-hydroxyethyl methacrylate)
PILs: poly­(ionic liquids)
*photo*ATRP: photoinduced ATRP
PMDETA: *N*,*N*,*N*′,*N*″,*N*″-pentamethyldiethylenetriamine
PMETAC: poly­[2-(methacryloyloxy)­ethyl trimethylammonium chloride]
PMOXA: poly­(2-methyl-2-ozazoline)
PMOXA-MA: poly­(2-methyl-2-oxazoline) methacrylate macromonomer
PNAPMAM: poly­(*N*-3-(aminopropyl)­methacrylamide)
PNIPAM: poly­(*N*-isopropylacrylamide)
POEGMA: poly­(oligo­(ethylene glycol) methyl ether methacrylate)
POEGMA_188_: poly­(di­(ethylene glycol) methyl ether methacrylate)
PPEGMA: poly­(poly­(ethylene glycol) methyl ether methacrylate)
PSPMA: poly­(3-sulfopropyl methacrylate potassium salt)
QPDMAEMA: quaternized poly­(2-(dimethylamino)­ethyl methacrylate)
RAFT: reversible addition–fragmentation chain transfer
RDRP: reversible deactivation radical polymerization
RH: relative humidity
RM: residual moisture
ROS: reactive oxygen species
RT: room temperature
Ru­(bpy)_3_: ruthenium tris­(2,2’-bipirydine)
saATRP: sacrificial-anode ATRP
SARA: supplemental activator and reducing agent
SBMA: sulfobetaine methacrylate
SCFS: single-cell force spectroscopy
*se*/*e*ATRP: electrochemically mediated ATRP
SF: survival fraction
SI-ATRP: surface-initiated atom transfer radical polymerization
SI-RAFT: surface-initiated reversible addition–fragmentation chain transfer
SI-RDRP: surface-initiated reversible deactivation radical polymerization
SMCs: human aortic smooth muscle cells
SNP: single nucleotide polymorphism
TCPS: tissue culture polystyrene
*T*_g_: glass transition temperature
THF: tetrahydrofuran
*T*_m_: thermal melting temperature
TPMA: tris­(2-pyridylmethyl)­amine
UCMSC: umbilical cord-derived mesenchymal stem cells
UV: ultraviolet
VBBI^+^Tf_2_N^–^: 1-(4-vinylbenzyl)-3-butylimidazolium bis­(trifluoromethylsulfonyl)­imide]
vWF: von Willebrand factor
WCA: water contact angle
